# Supplementary material for: Urine and Free Immunoglobulin Light Chains as Analytes for Serodiagnosis of Hantavirus Infection
Source: Viruses. 2019 Sep 1;11(9):809. doi: 10.3390/v11090809 (PMC6783946; doi:10.3390/v11090809)
Supplement: Supplementary file 1 [file viruses-11-00809-s001.zip › Supplementary Figure caption.docx]

Supplementary Figure caption

**Figure S1**: uLFRET titration series. This figure shows the uLFRET score from serially diluted pooled positive and negative samples. The negative counts at the higher dilutions reflect the lack of calibration for the lower concentrations.

**Figure S2:** uLFRET and pLFRET value timelines in individual patients. Samples from ten patients were selected to demonstrate the development of the (normalized signal/background) uLFRET and pLFRET scores over time. In all charts the title is the patient’s pseudonym, the y-axis represents the LFRET score, and the x-axis represents the sampling time (in days after onset of fever). The uLFRET scores are in yellow and the pLFRET scores are in red.

**Figure S3.** uLFRET and pLFRET scores of parallel samples compared. The (signal/background) uLFRET and pLFRET scores across the entire sample set plotted together. For clarity, five outliers were removed before generating the graph.
