# Supplementary figures and images for: Urine and Free Immunoglobulin Light Chains as Analytes for Serodiagnosis of Hantavirus Infection
Source: Viruses. 2019 Sep 1;11(9):809. doi: 10.3390/v11090809 (PMC6783946; doi:10.3390/v11090809)

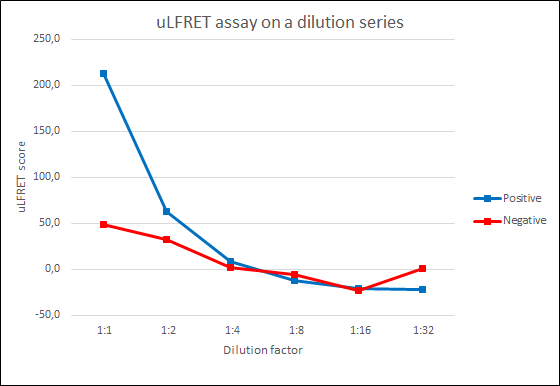

Supplement: Supplementary file 1 [file viruses-11-00809-s001.zip › Figure S1.tif]

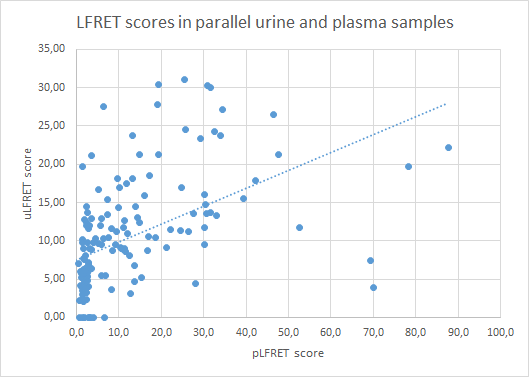

Supplement: Supplementary file 1 [file viruses-11-00809-s001.zip › Figure S2.tif]
